# The potential role of aging on acute phase proteins and hypotalamus-pituitary-adrenal axis: A representative cohort study in pregnant mares

**DOI:** 10.1016/j.vas.2026.100616

**Published:** 2026-03-07

**Authors:** Deborah La Fauci, Pietro Medica, Esterina Fazio, Giuliana Barbiera, Maria Gemma Velasco-Martinez, Katiuska Satué

**Affiliations:** a Department of Veterinary Sciences, Veterinary Physiology Unit, Polo Universitario Annunziata, Via Palatucci 13, 98168 Messina, Italy; b Pharmaceutical and Chemical Technician, 98168 Messina, Italy; c Department of Animal Medicine and Surgery, Faculty of Veterinary Medicine, CEU-Cardenal Herrera University, Tirant lo Blanc, 7, Alfara del Patriarca, 46115 Valencia, Spain

**Keywords:** SAA, Hp, CRP, ACTH, Cortisol, Pregnant mares, Age

## Abstract

Pregnancy in mares triggers physiological adaptations involving changes in inflammatory biomarkers, particularly acute-phase proteins (APPs), and the activation of the hypothalamic–pituitary–adrenal (HPA) axis. However, the extent to which these responses are influenced by maternal age remains unclear. The hypothesis of this study was that both APP profile and HPA axis activity during gestation are modulated by the age of the mare. Accordingly, the objective was to evaluate these variables throughout pregnancy and assess the impact of age on their physiological regulation. A total of 41 Spanish Purebred mares were evaluated: n. 31 were pregnant (n. 15 of ≤10 years; n. 16 of >10 years old) and n. 10 were non-pregnant mares. Pregnant mares were monitored monthly throughout gestation, to assess temporal changes in APPs and HPA axis activity. Blood samples were collected to measure serum amyloid A (SAA), haptoglobin (Hp), C-reactive protein (CRP), adrenocorticotropic hormone (ACTH), and cortisol (CORT) concentrations. Statistical analysis included repeated measures ANOVA, and *t*-tests to assess biomarker differences by age and pregnancy status. Pregnant mares, particularly older individuals, showed elevated concentrations of inflammatory and endocrine markers compared to non-pregnant ones (*p* < 0.05). These findings demonstrate that pregnancy significantly alters inflammatory and endocrine biomarkers in mares; therefore, older pregnant individuals showed more pronounced and sustained increases compared to both younger pregnant and non-pregnant mares. This suggests that maternal age influences the physiological adaptation to pregnancy, particularly in the regulation of the HPA axis and acute-phase response.

## Highlights

• Serum amyloid A, haptoglobin, C-reactive protein, ACTH, and cortisol were longitudinally monitored throughout gestation.
• Pregnancy induces measurable changes in acute-phase proteins and HPA axis activity, with maternal age effects.
• Older mares display heightened and prolonged inflammatory and endocrine responses compared to younger counterparts.
• Findings identify age as a critical factor influencing physiological adaptation during equine pregnancy.

## 1 Introduction

The interacting responses of the endocrine and immune systems characterize various forms of stress. Pregnancy triggers a complex interplay of physiological processes, including the activation of inflammatory pathways—such as the release of acute-phase proteins (APPs)—and the stimulation of the hypothalamic–pituitary–adrenal (HPA) axis. Neuroendocrine-immune system responses to stress are characterized by multiple checks and balances and interacting feedback loops. This orchestrated process, usually referred to as “stress response”, involves various mechanisms that allow the body to make the necessary physiological and metabolic adjustments required to cope with the demands of homeostatic challenge (Schradin et al., 2023). It was hypothesized that steroids released into the circulation from adrenal cortex contribute to stress resistance, but may also be responsible for pathological changes (Herman, 2022). In general, changes of the immune status reflect an animal’s reaction to a stressful stimulus (Silva & Gomes, 2024). A close correlation between inflammatory markers and the HPA axis has long been widely demonstrated in women, in which the dysregulation of this axis occurred in turn alternatively by some inflammatory markers like CRP, and with advancing age (Tolmay et al., 2012).

The possible linkage between acute stress response and immune status as dynamic adaptive response in sport horses was recently assessed (Arfuso et al., 2022).

Therefore, the profile of APPs has been evaluated in cyclic and pregnancy mares, showing their limited increase during pregnancy, independent of the hormonal dynamics, although the age was not considered (Satué et al., 2021). The same authors have shown that it is possible to use the APPs in breeding Spanish Purebred mares, considering other comparative equine breeds, as reliable diagnostic tools to assess both healthy status, and/or the development of subclinical reproductive inflammatory processes (Satué et al., 2021). APPs concentrations were greater in old horses than young ones, suggesting that the horses’aging may have induced a peak of acute phase responses (APR) meaning the greatest inflammation, compared to young horses (Arfuso et al., 2023; Herbst et al., 2022). In addition, although serum C-reactive protein (CRP) evaluation cannot be used for specific diagnosis of disease, it is considered a reliable and sensitive test able to give consistent information on the horses’ general clinical state of health (Yamashita et al., 1991).

Another finding of interest is the adaptive responses of the maternal HPA axis during pregnancy and lactation, inducing an its attenuated or increased stress reactivity response both in women (Altemus et al., 1995; de Weerth & Buitelaar, 2005) and in female rodents (Brunton et al., 2008; Fischer et al., 1995; Ma et al., 2005; Neumann et al., 1998; Walker et al., 2001), showing the naturally reversible hypercorticalism plasticity in the mechanisms governing HPA activity. Moreover, pregnancy and early post-natal period represent the greatest vulnerable phases of ontogenesis, programming the HPA axis’ disruptions of adulthood (Long et al., 2010; Renard et al., 2010; Schmidt et al., 2009) and aging (Goncharova, 2009; Solas et al., 2010); hence, this reduced HPA axis response to stressors represents an apparent adaptive response, providing a consistent and vital fetal and neonatal protection from an excess of toxic glucocorticoid concentrations in later life. What is more, the greatest hyperactivation of the HPA axis normally occurs in aging subjects, with an increase in the variability of HPA axis dysregulation, greater in humans and non-human primates than in rodents (Goncharova, 2013)**.**

In pregnant mares an increase of adrenocorticotrophic hormone (ACTH) and cortisol (CORT) concentrations was also described (Fazio et al., 2009; Marcilla et al., 2017; Satué et al., 2011); it was probably responsible for the influence of the placental development, the proper achievement of pregnancy and foetal viability, metabolism and growth (Martin & Crump, 2003). Neverthless, in a large number of researches, the maternal age is not controlled for parity, inducing consequently significant bias, nor a clear correlation between immune reactivity and HPA axis response was recorded**.**

Our interest in the maternal inflammatory response during physiological pregnancy stems from the observation that aging may alter the activation of inflammatory biomarkers and of HPA activity throughout the entire gestation. Therefore, this study aimed to compare the gestational profiles of APPs and HPA axis hormones in young and aged mares to identify age-related differences in physiological adaptation to pregnancy.

## 2 Materials and methods

This study used and applied methodology and procedures in total agreement with the guidelines of the Spanish law (RD 37/2014) regulating the protection of animals used for scientific purposes. The Animal Ethics Committee for the Care and Use of Animals of the CEU-Cardenal Herrera University (Spain) stated that this present study did not required any ethical approval, as it did not qualified as an animal experiment subjected to the Spanish law.

### 2.1 Animals and housing

A total of 41 Spanish Purebred mares were included in the study. 31 were pregnant mares and were classified into two age groups: younger pregnant mares (4 to 9 years or < 10 years; *n* = 15) and older pregnant mares (10 to 17 years or > 10 years; *n* = 16). The control group comprised 10 non pregnant mares age-matched to the study population: 5 mares <10 years and 5 mares ≥10 years.

All animals had normal reproductive histories, with previous deliveries resulting in live foals, and no recent pharmacological treatments or systemic disorders. Mares were housed in individual straw-bedded stalls (3.5 × 3.5 m) and had free access to natural pasture during daylight hours. The study was conducted at a single breeding facility located in Valencia, Spain (39.59° N, –0.40° W; altitude ∼35 m), under natural photoperiod and environmental conditions. To minimize handling-related variability, daily routines and personnel were kept constant across groups throughout the study.

### 2.2 Feeding and management

All mares were maintained under identical feeding and management conditions. Animals were fed twice daily at 08:00 and 20:00 h. Each mare received 2–3 kg of concentrate per meal (total 4–6 kg/day), and 2–3 kg/day of alfalfa hay with additional wheat straw offered to satisfy fiber requirements; rations were adjusted to individual needs. Water was available ad libitum, and mares had continuous access to pasture during daylight hours as well as to a salt block throughout the day. Body condition scores (BCS) ranged from 7 to 8 in both pregnant and non-pregnant mares, ensuring comparable nutritional and metabolic status across groups (Henneke et al., 1983). Husbandry practices and feeding schedules were identical across groups.

### 2.3 Breeding procedures (pregnant mares)

Pre-ovulatory status was monitored by transrectal ultrasonography using a 5- MHz probe (Sonosite 180 Plus) to evaluate follicular enlargement and uterine edema and to predict ovulation. When the dominant follicle reached ≥35 mm, mares received 1500 IU of hCG intramuscularly (Chorulon, Intervet). Artificial insemination was performed ∼30 h later using cooled semen from stallions at the Spanish State Stallion Depot (Zaragoza, Spain). A 60 mL insemination dose containing 300 × 10⁶ spermatozoa was deposited in the uterine body for each mare. Ovulation was verified by ultrasonography at 48 h post-insemination, and the absence of intrauterine inflammatory fluid was confirmed on day +5 after ovulation. All mares were bred once, except two that required a second insemination to achieve pregnancy. Pregnancy was confirmed via transrectal ultrasonography on day 16 post-ovulation and subsequently monitored longitudinally from early gestation through the 11th month, with mares undergoing regular clinical, hematological, and biochemical evaluations. Non-pregnant mares were not subjected to sham insemination, in order to avoid introducing procedure-related inflammatory or endocrine effects in the control group. These mares were sampled once during the breeding season (February–June), which limits control for potential seasonal variation in biomarker levels and is acknowledged as a study limitation.

### 2.4 Blood samples

The total duration of the study was 12 months, from February 2020 to February 2021, allowing for consistent time-course monitoring throughout gestation. The follow-up period in pregnant mares extended from day 16 post-conception following ultrasonographic confirmation of pregnancy until approximately 15 days prior to parturition, covering nearly the entire gestational timeline. Monthly sampling intervals were strategically selected to capture key physiological transitions associated with pregnancy progression. The duration of pregnancy in these mares ranged from 330 to 345 days. All deliveries were uneventful, the foals were clinically normal, and no postpartum complications were observed. This longitudinal design enabled the characterization of temporal patterns in APPs and HPA axis activity, both of which are known to undergo gradual modulation throughout gestation. In non-pregnant mares, blood samples were collected during estrus, coinciding with the timing of the first sampling in the pregnant group, to provide a baseline reference under comparable physiological and environmental conditions.

Blood collection was performed via jugular venipuncture using 20 mL Luer-lock disposable syringes (Becton Dickinson Discardit® II) attached to 40 mm, 18–20 G needles (Sterican®, Braun Melsungen AG). To minimize handling-induced variability in ACTH and CORT, all sampling was performed by the same two trained technicians between 08:00 and 11:00 h, after ≥20 min of quiet standing in the stall, and without sedation. Each sample was transferred into glass tubes containing clot activators and PS granules for serum collection (Tapval®), refrigerated at 4 °C for transport, and subsequently centrifuged at 3500 rpm for 10 min (P Selecta® Centrifuge). The resulting serum was stored at −20 °C until analysis.

### 2.5 Circulating serum amyloid type (SAA), haptoglobin (Hp), C-reactive protein (CRP), adrenocorticotropic hormone- (ACTH), and cortisol (CORT) determinations

Serum SAA was quantified by automated immunoturbidimetry on a Cobas Mira Plus using the monoclonal latex-enhanced VET-SAA (Eiken) reagent, which has been validated in horses (Jacobsen et al., 2019). with a limit of detection of ∼1.2 mg/L, a broad working range from 0 to >6000 mg/L, and intra- and inter-assay CVs below ∼10–12% within the 0–3,000 mg/L interval.

Serum Hp concentrations (mg/L) were quantified using the PHASE Haptoglobin assay (Tridelta Development Ltd., Ireland) on a microplate reader. In equine analytical evaluations, Hp immunoassays show excellent precision, with within-run CVs ∼1.8–3.1% and between-run CVs ∼1.2–2.8%, and verified linearity across clinically relevant ranges (e.g., ∼7–122 mg/dL); accordingly, precision <5% is expected in the concentration range encountered in this study (Contreras-Aguilar et al., 2018).

Serum C-reactive protein (CRP) was quantified by latex-enhanced immunoturbidimetry on a Cobas Mira Plus using Randox reagents; in horses, turbidimetric CRP methods show linearity of approximately 1–400 mg/L with limited interference from bilirubin, hemoglobin, and lipids, and within this range assay imprecision is typically <5%

Endogenous ACTH (pg/mL) was quantified by a commercial radioimmunoassay (Phoenix Pharmaceuticals, Burlingame, CA, USA) previously applied and validated in pregnant Spanish Purebred mares (Marcilla et al., 2017). Serum cortisol (CORT, ng/mL) was measured by a competitive immunoassay validated for equine plasma and likewise used in that cohort. For equine cortisol immunoassays, reported analytical performance includes intra-assay CVs of ∼7–11%, inter-assay CVs of ∼9–11%, and sensitivities around ∼1 ng/mL.

### 2.6 Statistical analysis

Serum concentrations of SAA, Hp, CRP, ACTH and CORT were compared across three independent groups: females pregnant <10 years, females pregnant ≥10 years, and non-pregnant females. Data were screened for outliers and assessed for normality using the Shapiro–Wilk test and for homogeneity of variances using Levene’s test. When assumptions of normality and homoscedasticity were met (or improved after log-transformation when appropriate), group differences were evaluated using one-way ANOVA. To account for potential confounding effects, age (in years) and month of gestation were included as covariates in an ANCOVA model for pregnant females. When parametric assumptions were not met, even after transformation, the Kruskal–Walli’s test was applied. Pairwise comparisons were performed using Tukey’s post hoc test for ANOVA models and Dunn’s test with Bonferroni correction for non-parametric analyses.

Correlations among physiological parameters (AAS, Hp, CRP, ACTH and CORT) were examined using Pearson’s coefficients for normally distributed variables and Spearman’s rank coefficients for non-normal variables. Partial correlations controlling for age and gestational months were also computed. Statistical significance was set at *p* < 0.05.

## 3 Results

Given the sample size and the magnitude of the observed effects, the statistical power of the study was sufficient to detect significant differences between groups. This supports the robustness of the findings and minimizes the likelihood of Type II errors. Table 1, Table 2 present the mean ± standard deviation (SD) of circulating concentrations of SAA, Hp, CRP, ACTH, and CORT in pregnant mares of different ages. Significant month-to-month differences were observed throughout the 11 months (mo.) of gestation in both age groups.

**Table 1:** Circulating serum amyloid A (SAA), haptoglobin (Hp) and C-reactive protein (CRP), adrenocortico-trophic hormone (ACTH), and cortisol (CORT) concentrations (Mean ± SD) in < 10 years pregnant Spanish Purebred mares throughout the entire pregnancy. Letters indicate significant differences (*p* < 0.05) vs other months (mo.): *a* = vs 7–11 mo.; *b* = vs 8, 9, 11 mo.; *c*= vs 6–11 mo.; *d*= vs 5,6,8 mo.; *e*= vs 8,10,11 mo.; *f*= vs 5,7 mo.; *g*= vs 6,8,10 mo.; *h*= vs 3,8 mo.; *i*= vs all mo.

Pregnant mares of < 10 years					
Month of pregnancy	SAA (mg/dL)	Hp (mg/L)	CRP (g/dL)	ACTH (pg/mL)	CORT (ng/mL)
**1**	0.02 ± 0.01 (0.01–0.04)	1.90 ± 1.19a (0.8–4.2)	16.8 ± 4.11d (12.2–22.5)	5.76 ± 3.52 (2.1–12.3)	37.4 ± 4.42l (30.5–44.1)
**2**	0.02 ± 0.01 (0.01–0.03)	1.75 ± 0.65b (0.9–2.8)	9.51 ± 6.22e (3.0–18.7)	4.61 ± 3.19 (1.2–10.5)	35.9 ± 2.53m (32.0–39.5)
**3**	0.03 ± 0.07 (0.01–0.15)	1.05 ± 0.70c (0.4–2.3)	15.4 ± 6.91f (7.2–26.8)	4.89 ± 2.44 (2.1–9.3)	38.5 ± 3.47m (33.2–44.1)
**4**	0.11 ± 0.01 (0.09–0.13)	2.06 ± 0.46a (1.4–2.9)	9.78 ± 5.23i (4.1–18.2)	6.14 ± 3.29 (2.5–11.4)	32.9 ± 3.63c (27.8–38.6)
**5**	0.11 ± 0.01 (0.09–0.13)	2.78 ± 0.76 (1.6–4.1)	5.96 ± 3.97 (2.0–12.3)	13.6 ± 5.12 (7.4–21.5)	39.2 ± 2.20 (36.1–42.5)
**6**	0.01 ± 0.05 (0.01–0.12)	2.90 ± 1.17 (1.4–4.8)	3.90 ± 2.60 (1.2–8.3)	3.35 ± 3.23 (0.9–9.1)	37.6 ± 4.43 (31.2–44.3)
**7**	0.08 ± 0.06 (0.02–0.18)	4.18 ± 0.46 (3.5–4.9)	6.40 ± 7.02 (1.1–18.9)	12.2 ± 8.08 (3.4–25.6)	34.8 ± 3.94 (29.5–40.2)
**8**	0.19 ± 0.01 (0.17–0.21)	3.42 ± 0.58 (2.6–4.3)	8.37 ± 8.48 (1.2–22.5)	4.14 ± 2.57 (1.3–8.9)	34.0 ± 3.94 (28.7–39.6)
**9**	0.13 ± 0.05 (0.07–0.22)	4.50 ± 0.75 (3.3–5.9)	11.2 ± 7.56 (3.4–23.1)	7.26 ± 3.28 (3.1–13.2)	35.2 ± 1.41 (33.1–37.6)
**10**	0.13 ± 0.01 (0.11–0.15)	4.67 ± 0.55 (3.9–5.6)	10.2 ± 9.08 (2.1–24.3)	4.64 ± 2.09 (2.1–8.1)	35.4 ± 1.70 (32.9–37.8)
**11**	0.12 ± 0.01 (0.10–0.14)	3.51 ± 0.69 (2.6–4.7)	7.00 ± 5.47 (2.3–15.1)	7.08 ± 3.73 (2.9–13.4)	36.0 ± 2.12 (32.8–39.1)

**Table 2:** Circulating serum amyloid A (SAA), haptoglobin (Hp) and C-reactive protein (CRP), adrenocortico-trophic hormone (ACTH), and cortisol (CORT) concentrations (Mean ± SD) in > 10 years pregnant Spanish Purebred mares throughout the entire pregnancy. Letters indicate significant differences (*p* < 0.05) vs other months (mo.): *a* = vs 7–11 mo.; *b* = vs 8, 9, 11 mo.; *c*= vs 6–11 mo.; *d*= vs 5,6,8 mo.; *e*= vs 8,10,11 mo.; *f*= vs 5,7 mo.; *g*= vs 6,8,10 mo.; *h*= vs 3,8 mo.; *i*= vs all mo.

Pregnant mares of > 10 years					
Month of pregnancy	SAA (mg/dL)	Hp (mg/L)	CRP (g/dL)	ACTH (pg/mL)	CORT (ng/mL)
**1**	0.01 ± 0.01 (0.00–0.03)	1.71 ± 0.73c (0.25–3.17)	7.00 ± 5.47f (3.94–18.0)	7.08 ± 3.73h (0.38–13.54)	36.0 ± 1.67m (32.7–39.3)
**2**	0.05 ± 0.09 (0.13–0.23)	1.34 ± 0.41d (0.58–2.16)	11.1 ± 5.91f (5.72–17.2)	3.78 ± 1.07i (1.64–5.92)	36.9 ± 1.11n (34.7–39.1)
**3**	0.14 ± 0.01a (0.12–0.16)	0.99 ± 0.91e (0.83–2.81)	12.4 ± 6.00 *g* (6.4–18.4)	4.14 ± 1.43l (1.28–7.00)	38.3 ± 2.26o (33.8–42.8)
**4**	0.16 ± 0.02 (0.12–0.20)	0.62 ± 0.51c (0.40–1.54)	19.0 ± 7.20 *g* (11.2–23.4)	6.02 ± 1.65l (3.72–7.91)	45.4 ± 8.60p (38.2–52.6)
**5**	0.17 ± 0.03b (0.11–0.23)	1.28 ± 0.91 (0.54–3.10)	5.00 ± 3.97 *g* (1.94–9.21)	14.7 ± 7.01l (6.70–22.1)	37.8 ± 6.90q (29.7–44.0)
**6**	0.20 ± 0.17 (0.14–0.44)	2.93 ± 1.08 (0.77–5.09)	7.30 ± 6.97 (6.64–16.1)	9.75 ± 6.03 (2.31–17.2)	32.6 ± 7.15 (24.8–40.1)
**7**	0.23 ± 0.04 (0.15–0.31)	3.00 ± 1.26 (0.48–5.52)	15.8 ± 7.02 (7,03–27.0)	14.6 ± 4.04 (9.61–19.3)	38.5 ± 3.22 (32.1–44.0)
**8**	0.24 ± 0.05 (0.14–0.34)	4.40 ± 1.32 (1.76–7.04)	25.1 ± 10.9 (3.3–37,2)	5.77 ± 3.90 (2.03–13.6)	33.4 ± 3.45 (26.5–40.3)
**9**	0.30 ± 0.21 (0.12–0.72)	3.23 ± 0.95 (1.33–5.13)	16.7 ± 7.12 (2.5–30.9)	10.8 ± 6.23 (1.7–23.3)	34.1 ± 2.90 (28.3–39.9)
**10**	0.19 ± 0.02 (0.15–0.23)	4.27 ± 1.03 (2.21–6.33)	12.7 ± 10.2 (7.7–33.1)	5.52 ± 5.00 (4.5–15.5)	37.4 ± 5.10 (27.2–47.6)
**11**	0.21 ± 0.05 (0.11–0.31)	3.57 ± 0.77 (2.03–5.11)	9.70 ± 6.61 (3.5–22.9)	5.45 ± 2.36 (0.73–10.2)	36.6 ± 4.19 (28.2–45.0)

**Fig. 1:**
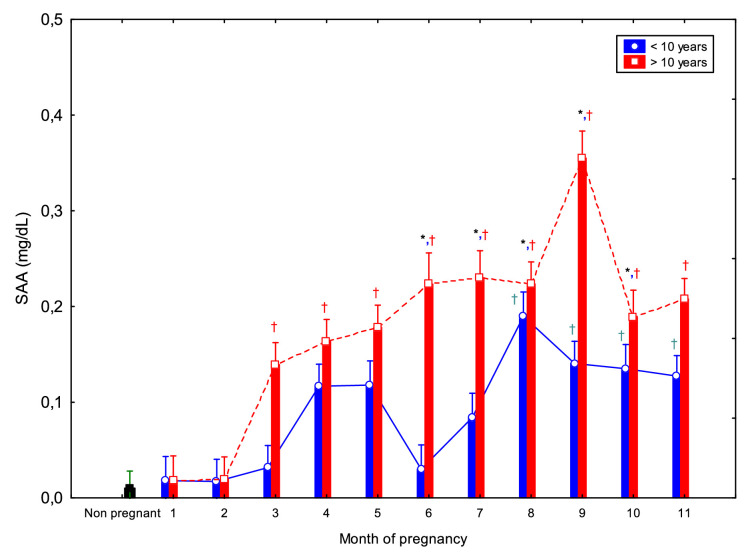
Circulating serum amyloid A (SAA) concentrations (Mean ± SD) in non-pregnant and pregnant Spanish Purebred mares aged <10- and >10-year-old throughout the entire pregnancy. Asterisk indicates significant differences between age groups (<10 vs. >10 years); ^†^ indicates significant differences versus non-pregnant mares (*p* < 0.05).

**Fig. 2:**
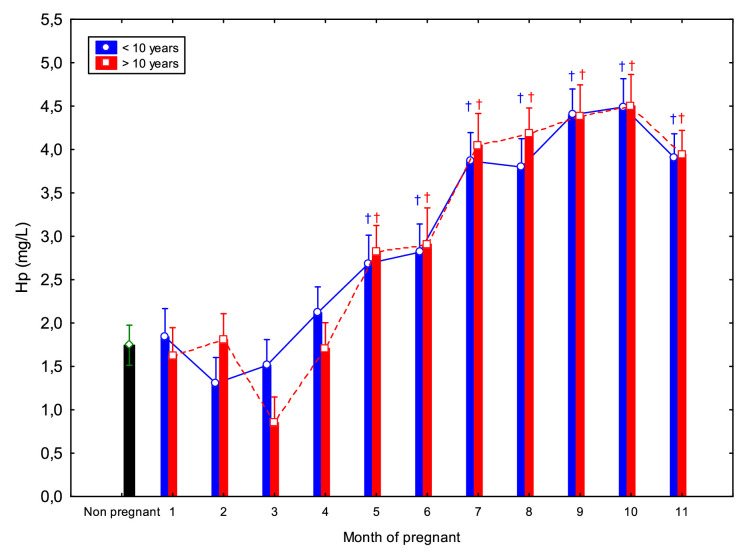
Circulating haptoglobin (Hp) concentrations (Mean ± SD) in non-pregnant and pregnant Spanish Purebred mares aged < 10- and > 10-year-old throughout the entire pregnancy. Mares aged 〈 10- versus mares 〉 10-year-old: non significative. ^†^ Indicates significant differences versus non-pregnant mares (*p* < 0.05).

**Fig. 3:**
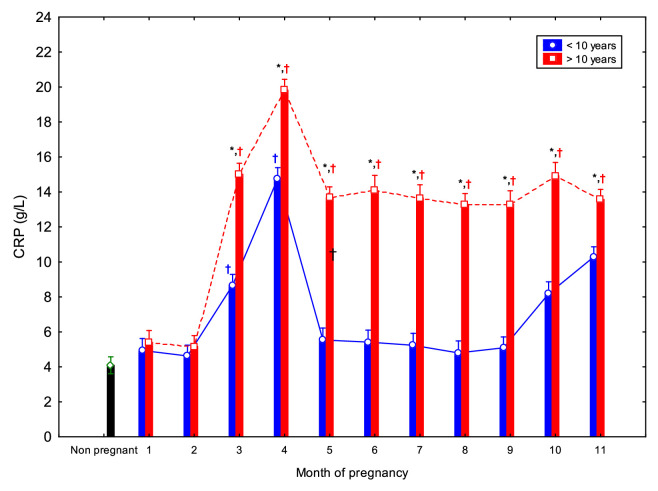
Circulating C-reactive protein (CRP) concentrations (Mean ± SD) in non-pregnant and pregnant Spanish Purebred mares aged <10- and >10-year-old throughout the entire Asterisk indicates significant differences between age groups (<10 vs. >10 years); ^†^ indicates significant differences versus non-pregnant mares (*p* < 0.05).

**Fig. 4:**
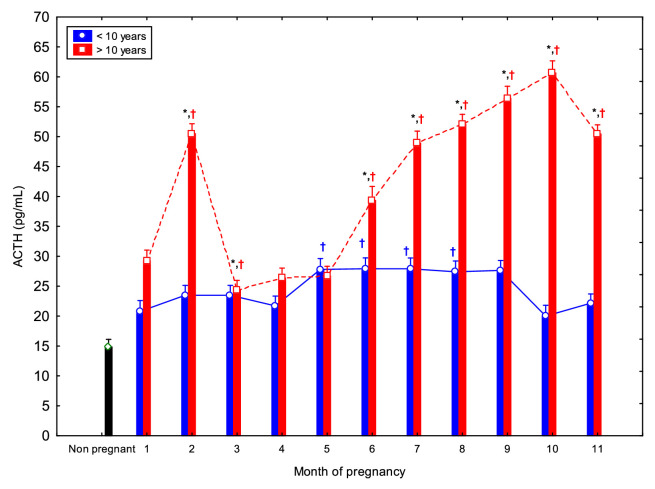
Circulating adrenocorticotrophic hormone (ACTH) concentrations (Mean ± SD) in non-pregnant and pregnant Spanish Purebred mares aged <10- and >10-year-old throughout the entire pregnancy. Asterisk indicates significant differences between age groups (<10 vs. >10 years); ^†^ indicates significant differences versus non-pregnant mares (*p* < 0.05).

**Fig. 5:**
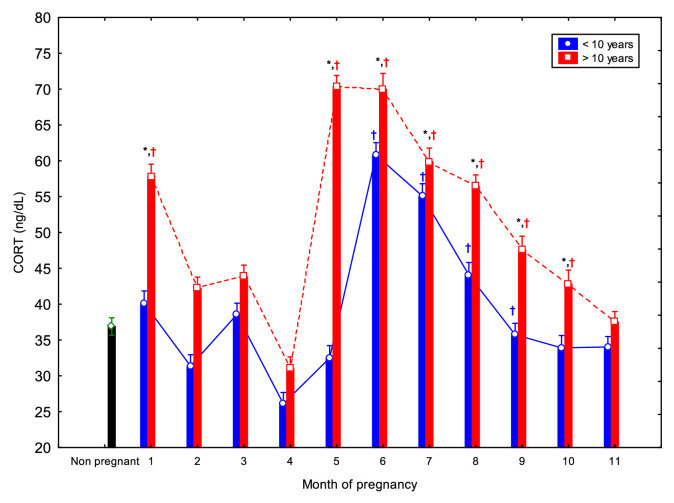
Circulating cortisol (CORT) concentrations (Mean ± SD) in non-pregnant and pregnant Spanish Purebred mares aged <10- and >10-year-old throughout the entire pregnancy. Asterisk indicates significant differences between age groups (<10 vs. >10 years); ^†^ indicates significant differences versus non-pregnant mares (*p* < 0.05).

The comparison between young and old mares throught the entire pregnancy for all parameters is shown in Figs. 1–5.

SAA concentrations exhibited a gradual two-phase pattern, with stable low values during early gestation (1 to 3 mo.) followed by a sustained increase from 4 mo. to term (Fig. 1). Specifically, SAA concentrations at 3 mo. were significantly lower than those at 7 to 9 mo., and concentrations at 5 mo. were lower than at 9 mo. (*p* < 0.05) (Table 1, Table 2).

Compared to non-pregnant, mares over 10 years showed significantly higher SAA concentrations from 3 to 11 mo., while younger mares (<10 years) exhibited significant differences from 8 to 11 mo. Additionally, older mares had higher SAA concentrations than younger ones between 6 and 10 mo. of gestation (*p* < 0.05; Fig. 1).

Hp concentrations in younger and older mares were significantly reduced during early gestation (1 to 3 mo.), increasing progressively throughout mid and late pregnancy (7 to 10 mo.; *p* < 0.05). From 4 mo. onward, a progressive increase in Hp concentrations was observed (Table 1, Table 2). Specifically, concentrations at 1 mo. were significantly lower than those at 7 to 10 mo.; at 2 mo., lower than at 7, 9, and 10 mo.; at 3 mo., lower than at 6 to 10 mo.; and at 4 mo., lower than at 7 to 10 mo. (*p* < 0.05). Compared to non-pregnant mares, Hp concentrations were significantly elevated from 5 to 11 mo. in both age groups (>10 and <10 years). No significant differences were detected between younger and older mares (Fig. 2).

CRP concentrations showed a biphasic pattern in both younger and older mares (Fig. 3). In mares over 10 years, CRP concentrations from 3 to 11 mo. were significantly higher than those observed in non-pregnant (*p* < 0.05). In younger mares (<10 years), significant differences compared to controls were found at 3 and 4 mo. Within-group comparisons revealed that younger mares had lower CRP concentrations at 1 mo. compared to 3, 4, and 10–11 mo. (*p* < 0.05); at 2 mo. compared to 4, 5, and 10–11 mo.; and at 3 mo. compared to months 4, 10, and 11 mo. Additionally, CRP concentrations at 4 mo. were significantly higher than those recorded from 5 to 11 mo. (Table 1). In older mares, CRP concentrations at 1 and 2 mo. were significantly lower than those from 3 to 11 mo. (*p* < 0.05). Moreover, CRP concentrations at 3 to 5 mo. were significantly higher than those at 8 to 11 mo. (Table 2). Overall, older mares exhibited consistently higher CRP concentrations from 3 to 11 mo., compared to younger mares (Fig. 3).

ACTH concentrations displayed distinct temporal patterns in older and younger mares (Fig. 4). In mares over 10 years, ACTH concentrations at 2, 3, and from 6 to 11 mo. were higher than those observed in non-pregnant (*p* < 0.05). In younger mares (<10 years), significant differences compared to controls were found between 5 and 8 mo. Within-group comparisons revealed that younger mares had lower ACTH concentrations at 1 mo. compared to 2, 3, and 5 to 11 mo. (*p* < 0.05); at 2 and 3 mo. compared to 5 to 11 mo.; and at 4 mo. compared to 6 to 11 mo. (Table 1). In older mares, ACTH concentrations at 1 mo. were lower than those at 2 and 7 to 11 mo. (*p* < 0.05). Additionally, ACTH concentrations at 2 mo. were higher than at 3 to 6 and 8 to 10 mo., while concentrations from 3 to 5 mo. were lower than those from 6 to 11 mo. (Table 2). Overall, older mares exhibited significantly higher ACTH concentrations at 1 and 2 mo., and from 6 to 11 mo., compared to younger mares (Fig. 4).

CORT concentrations exhibited a biphasic pattern in both younger and older mares (Fig. 5). In subjects over 10 years, concentrations at 2 mo. and from 5 to 10 mo. were higher than those in the non-pregnant group (*p* < 0.05), while in younger mares (<10 years), significant differences were observed from 6 to 9 mo. In younger mares, CORT concentrations at 1 mo. were higher than at 2, 4, and 5 mo. (*p* < 0.05); and concentrations at 2 and 3 mo. were higher than at 4 and 5 mo., but lower than those recorded from 6 to 11 mo.. Additionally, CORT concentrations at 4 mo. were significantly lower than those from months 6 to 10 mo. (Table 1). In older mares, CORT concentrations at 1 mo. were higher than at 2 and 4 mo. (*p* < 0.05), but lower than those from 5 to 11 mo. At 2 mo. values were significantly lower than those at 4 to 6 and 8 to 10 mo.; at 3 mo. values were lower than 6 to 8 mo.; and at 4 mo. values were lower than 5 to 11 mo.. Moreover, CORT concentrations at 5 mo. were higher than those from 6 to 9 mo. (Table 2). Overall, older mares exhibited significantly higher CORT concentrations at 1 mo. and from 5 to 10 mo. of pregnancy compared to younger mares (Fig. 5).

Circulating SAA concentration was positively correlated both with Hp (*r* = 0.51; *p <* 0.05) and ACTH (*r* = 0.38; *p <* 0.05) only in the oldest mares.

## 4 Discussion

### 4.1 Effect of pregnancy

According to literature comparing equine species, but also different animal species, often cited as an experimental model, the results described in the mares under study, especially in terms of SAA increase from 7 mo. until the end, are different. In fact SAA concentrations followed a biphasic pattern, with reduced concentrations during early pregnancy (1 to 3 mo.) and a gradual rise from 4 mo. through late gestation (5 to 11 mo.). This trend confirms changes of SAA recorded during the last four mo. of gestation both in Thoroughbred (Nunokawa et al., 1993) and Quarter Horse mares (Satoh et al., 1995). In proportion to the duration of pregnancy in the goat, an increase in SAA has been also described since the 2 mo., remaining elevated until the end of gestation and peaking at kidding (Czopowicz et al., 2017). What is more, the SAA increase until the end of pregnancy is corroborated by the knowledge that it generally increase in the peripartum period, induced by tissue damage during the passage of the fetus through the birth canal and the restoration of the endometrium (Coutinho da Silva et al., 2013). In the same way, the presence of human SAA in trophoblast at the 1st trimester (Kovacevic et al., 2006) and in placental cells at the 3rd trimester (Sandri et al., 2014), suggests that this protein may have a pivotal function during the advancement of the pregnancy, modulationing the IL-1β, IL-6 and IL-8 (Bowen et al., 2002; Osman, 2003) and metalloprotease involvements (Xu et al., 2002), triggering the onset of labor and delivery also in women. In addition, in woman the physiological 3rd trimester of pregnancy is characterized by significant peripheral blood leukocytes’ activation, with change in both SAA and CRP concentrations (Sacks et al., 1998).

Neverthless, results obtained in this study don’t conform data previously obtained in Spanish Purebred mares, but not groupeded by age (Satué et al., 2021), nor those recorded in bitches (Ulutas et al., 2009), in which no modifications were observed throughout gestation. Notably, SAA concentrations in pregnant mares, particularly during mid to late gestation, were consistently higher than those observed in non-pregnant mares, suggesting a pregnancy-associated inflammatory modulation.

Hp concentrations in pregnant mares were significantly greater from 4 mo. to the end of gestation, with peak concentrations observed between 7 and 10 mo. Therefore, these Hp increases during the late gestation, could be due to a close relationship between estrogens and Hp documented in mares during the last four months of pregnancy (Taira et al., 1992). Moreover, Hp concentrations during pregnancy were consistently elevated compared to those in non-pregnant mares, suggesting a gestation-related upregulation of this APP. These findings partially align with data reported in bitches and women. Indeed, in pregnant bitches, the Hp increase occured from the 3rd week, concurrent with the LH increase, subsequent to the embryo implantation and the placentation, and remaining greates until the delivery; for this reason, Hp can be considered a source for early pregnancy diagnosis in this species (Vannucchi et al., 2002).

In women, Hp exhibited a biphasic pattern, with two release peaks during the 1st and 3rd trimester, and a decrease observed at 24 weeks of gestation (Haram et al., 1983; Larsson et al., 2008), hypothesizing its placental synthesis (Berkova, 2001) with to the angiogenic role of the placenta and fetal development (Herrler et al., 2004). Indeed, a decrease of Hp concentration along pregnancy was described in women (Gatzka et al., 2002), as effects of hemodilution and estrogen, according to observed by experimental application of estrogen derivatives (Bicho et al., 2009).

On the other hand, a stable Hp concentration throughout gestation was described in pregnant sows (Sorrells et al., 2007), goats (Czopowicz et al., 2017), and in healthy mares at term of pregnancy, with normal foals, compared to mares with placentitis (Canisso et al., 2014).

The CRP patterns observed in this study are partially in agreement with previous findings observed in Thoroughbred mares, where concentrations declined during the last four months of gestation, reaching a minimum approximately two months before foaling (Yamashita et al., 1991). In fact, our results revealed significantly greater CRP concentrations during late pregnancy compared to non-pregnant mares, suggesting a possible species- or breed-specific modulation of inflammatory responses during gestation. Interestingly, another study conducted in mares of the same breed reported no significant changes in CRP concentrations throughout pregnancy (Satué et al., 2021), although age was not considered as a variable in that analysis.

Likewise, in bitches CRP increased significantly during pregnancy; indeed, this is due to the fact that by day 16 after the luteinizing hormone (LH) surge, the endometrial implantation sites were carried out, according to the release of cytokines, and consequently hepatic synthesis of APPs (Gruys et al., 2005). A lack of consistency regarding the CRP changes during pregnancy was described in women, since only 17.4 % manifested a progressive increase, 30 % described a progressive reduction, and the largest number of studies equal to the 52.2 % revealed fluctuations (Belo et al., 2005). In pregnant women, consistent and significant immunological alterations are highlighted as the maternal immune system encounters foreign antigens caming from the semi-allogeneic fetus and the placenta (Gómez-Chávez et al., 2021; Mor et al., 2017).

The immune system also is subject to change during developmental stages; hence, the 1st trimester is predominantly proinflammatory to facilitate blastocyst implantation, creating an inflammatory environment that is protective for the implanting embryo; the 2nd trimester of pregnancy, during which fetal growth occurs, is characterized by an anti-inflammatory Th2 environment so the immune response should be less intense; finally, during the 3rd trimester, a shift toward a pro-inflammatory Th1 response becomes necessary for fetal development (Mor et al., 2017), which could be identify with an increase in CRP concentrations in late pregnancy in older mares. Finally, the proinflammatory environment in the uterus reappears at the time of delivery (Gómez-Chávez et al., 2021).

Similar to what has been described in the bitch and woman, extrapolations can be made and physiological considerations drawn for the equine species as well.

On the other hand the profile described for ACTH along pregnancy confirmed partially its increase recorded in Spanish Purebred mares at 2 mo., but no that recorded at 3 mo., following by a decrease until values similar to those recorded at the beginning of the study at 4 mo. (Marcilla et al., 2017).

The results obtained in this study regarding the initial increase of serum ACTH concentrations at 2 mo. are partially in agreement with the data previously reported by Fazio et al. (2009); Satué et al. (2011) and Marcilla et al. (2017) in which this early increase was detected at 3 mo. of pregnancy. Moreover, in both cases it is possible to presume that the valuable explanations of this initial increase might be due to increased synthesis of placental cortisol-releasing hormone (CRH) from the beginning of the pregnancy, stimulating ACTH secretion and foetal-maternal CORT synthesis (Mastorakos & Ilias, 2003). However, it is shown that in pregnant mares circulating CRH concentrations did not appear to increase (Ellis et al., 1994). Specifically, available evidence suggests that endometrial cups are already present at day 35–40 of gestation in the mare and are able to modulate immunity; moreover, the chorionic junctions induce a suppression of lymphocyte proliferation, with successive expression of various cytokines (Flaminio & Antczak, 2005).

A large increase in circulating CORT described at 5 mo. of pregnancy compared with the first 4 mo., following by a decrease towards the values found at the beginning of the pregnancy, confirmed previous data recorded in Arabians (Gill et al., 1985), Standardbreds (Flisińska-Bojanowska et al., 1991), Thoroughbreds (Fazio et al., 2009), Spanish Purebred (Marcilla et al., 2017; Satué et al., 2011), and crossbred mares (Hicks et al., 2021).

CORT concentrations described in Spanish Purebred mares progressively increased from 1 mo., peaking at 4 and 5 mo. (Satué et al., 2011) and also between 5 and 8 mo. of pregnancy (Fazio et al., 2009). Based on these results, the HPA axis is activated during normal gestation, resulting in physiological hypercortisolism status (Haritou et al., 2008; Marcilla et al., 2017). Several mechanisms that explain this increase in CORT in the pregnant mare have been proposed: -increased plasma CRH in response to its placental production (Hoffman et al., 2003), which, in turn, stimulates ACTH secretion; -hypertrophy of the maternal adrenal glands due to their own ACTH secretion (Mastorakos & Ilias, 2003); -increased P_4_ in response to fetal adrenocortical activity (Fazio et al., 2009); and -placental transfer of CORT from the fetus to the mare (Nagel et al., 2012). In addition, the mare becomes insulin resistant at the end of gestation (George et al., 2007) in response to the insulin-like effects of CORT (Fowden et al., 1984) and functional modifications of pancreatic cells. Physiological insulin resistance develops in pregnant mares and is more pronounced at 7–8 mo. of pregnancy, when a marked fetal bone growth and an increase in mare body weight occurred (George et al., 2011); this induces elevated circulating glucose concentrations, preserving them for consumption in organs such as the brain, and improving the glucose transfer to placenta to meet fetal demands (Fowden et al., 2005), and to ensure adequate glucose uptake through the placenta to nourish the developing fetus (George et al., 2011).

However, CORT decreases from the 6th mo. (Fazio et al., 2009) or the 7th mo. (Satué et al., 2011), could be related to the effect of the estrogens, since the two parameters are negatively correlated (Fazio et al., 2009). Two weeks before foaling, Arfuso et al. (2021) reported an increase of approximately 200 % in CORT compared to non-pregnant mares. In this study, only basal concentrations of ACTH and CORT were measured, which provides a snapshot of endocrine status but does not constitute a full functional assessment of the HPA axis. Therefore, while our findings suggest age-related differences in hormone concentrations during pregnancy, further studies using stimulation or suppression tests are needed to confirm alterations in HPA axis responsiveness.

### 4.2 Effect of age

The changes of the inflammatory markers described in this study are in line with previous studies that have investigated the effect of age on inflammation status of horses (Siard-Altman et al., 2020; Yamashita et al., 1991), humans (Le Gall & Ardaillou, 2009), and rats (Ahmadian & Dabidi Roshan, 2018), corroborating the hypothesis that increased concentrations of certain key APPs are associated with age (Ahmadian & Dabidi Roshan, 2018; Le Gall & Ardaillou, 2009). APPs are involved in the initial non-adaptive (innate) immune response (Schaap et al., 2006). As tissue ages, the body experiences a form of chronic low-grade inflammation known as inflammaging, which tends to worsen over time. Inflammaging is thought to contribute to various age-related pathologies and physiological changes in aging tissues (Franceschi et al., 2000). Aging correlates with sustained immune activation, reflected in elevated inflammatory markers and immune cell engagement, increasing vulnerability to age-related conditions (Singh et al., 2024). This process is part of the immunosenescence phenomenon and involves normal, progressive fluctuations in the immune system, resulting in poor regulation or control of proinflammatory protein production during and after immune responses (Capri et al., 2006; Singh et al., 2024). Compared to younger horses, older horses exhibit increased production of proinflammatory cytokines by monocytes and lymphocytes (Herbst et al., 2022; Miller et al., 2021; Zak et al., 2020). Arfuso et al. (2023) showed a significant age-related difference in CRP concentrations in horses, parallel to the increasing trend of α1- and α2-globulins. This could reflect a more marked APR in adult and older horses than in young ones, as corroborated by the strong positive correlation found between CRP, α1- and α2-globulin concentrations and the age of the horses. These changes in inflammatory markers are consistent with those previously reported by Siard-Altman et al. (2020) in elderly horses, in which age was associated with changes in numerous inflammatory proteins, including CRP.

In addition to inflammaging, older horses experience age-related muscle wasting (Reed et al., 2015), like other species, including humans (Schaap et al., 2009). In longitudinal human studies, increased inflammatory markers, particularly tumor necrosis factor- α (TNF- α), Interleukin- 6 (IL-6), and CRP were correlated both with strength in the elderly and adecreased muscle mass (Schaap et al., 2009). These findings reflect a more raised APR concentrations in old horses than in young ones, as shown by the strong positive correlation found between the concentrations of some positive APPs, including SAA and Hp, and the age of pregnant mares.

In mares > 10 years of age, ACTH and CORT concentrations increased compared to those of young mares. Similar results have been reported in equines (Cavinder et al., 2007; Hart et al., 2016). It is known that advancing age leads to structural changes in the HPA axis; hypothalamic and pituitary cells become less sensitive to the negative feedback exerted by CORT, so that CORT does not inhibit the production of CRH and ACTH with the same efficacy. These effects have been reported in elderly equines subjected to prolonged stress. In fact, chronic stress desensitizes the HPA axis, making the negative feedback exerted by CORT less effective (Cappola et al., 2023; Stamou et al., 2023). Other health conditions, such as chronic inflammation or metabolic imbalances, may also contribute to elevated CORT concentrations in older horses (Sikorska et al., 2023). As would be expected, all these data indicate age-dependent differences between the responsiveness to pregnancy between young and old mares. It is possible to presume that defective stop signals could explain the exaggerated and/or prolonged HPA axis response to this physiological period in the old mares. The significant greater circulating ACTH—CORT concentrations in the aged mares suggested some resistance related effects for the negative feedback loop of circulating CORT on related hypothalamic glucocorticoid receptors.

However, these ideas are not supported by others. Indeed, in older horses, the degree of CORT binding to cortisol-binding globulin (CBG) or albumin is increased; ACTH concentrations do not increase sufficiently to stimulate adrenal CORT production; and the ability to replenish CORT due to greater experience in transport could be related to the lack of CORT elevation (Jacquay et al., 2023). Compared to 90 % of CORT bound to CBG and albumin (Alexander & Irvine, 1998; Hart et al., 2011), the remaining 10 % unbound is considered more biologically active and therefore available to bind to cytoplasmic steroid receptors, mediating most of the expected systemic effects of CORT (Stewart, 2008). However, aged mares may experience altered HPA axis function, leading to greater ACTH and CORT concentrations related with pituitary pars intermedia dysfunction (PPID), which involves an enlargement (hyperplasia) of the pituitary gland and increased ACTH secretion (Gehlen et al., 2020; McFarlane, 2011). Although none of the mares exhibited clinical signs compatible with PPID, the possibility of subclinical cases cannot be ruled out. Therefore, the observed increase in ACTH and CORT concentrations is interpreted as a physiological response potentially associated with age and pregnancy, but further diagnostic testing would be required to definitively exclude early-stage PPID.

These results clarify how maternal age influences endocrine and inflammatory dynamics during equine pregnancy. In older mares, sustained elevations in ACTH, CORT, SAA, and CRP point to prolonged activation of both the HPA axis and the acute-phase response. Conversely, younger individuals exhibited faster cortisol normalization and lower levels of acute-phase proteins, reflecting more efficient physiological regulation. These age-related contrasts emphasize the need for tailored monitoring strategies throughout gestation and suggest increased vulnerability to stress and inflammation in older mares.

The temporal patterns observed in SAA, Hp, CRP, and ACTH–CORT profiles appear to reflect physiological adaptations rather than pathological changes. Despite elevated concentrations of Hp and SAA and fluctuating CRP levels, ACTH and CORT remained within expected ranges in older mares, indicating preserved hormonal regulation. This interpretation is supported by statistically significant, albeit weak, correlations between APPs and HPA axis hormones. Such modest associations imply that additional factors—such as individual variability, environmental conditions, or other regulatory pathways—may contribute to the observed responses. Therefore, caution is warranted when interpreting these findings, and further research with larger cohorts and longitudinal designs is needed to clarify their biological significance.

## 5 Importance of the study, rationale, and future clinical applications

While an optimal and physiological expected inflammation in gestational tissues is an indispensable event of implantation and parturition processes (Sieg et al., 2022), our observations could have a practical application in this field, since an increase of SAA, Hp or CRP concentrations along the pregnancy could be facilitate an early diagnosis of aberrant inflammation in some insidious diseases, such as endometritis (Canisso et al., 2014; Tuppits et al., 2014), early embryonic mortality (Krakowski et al., 2011), and placentitis (Canisso et al., 2014; Coutinho da Silva et al., 2013).

The SAA assessment in mares with a history of pregnat high-risk can provide valuable and additional information, as soon as its concentration increases rapidly according to the ascending placentitis (Canisso et al., 2014). Following a diagnosis of placentitis, tracking SAA can help monitor risk of impending abortion (Canisso et al., 2014; Coutinho da Silva et al., 2013), since it increases on average 2–6 days prior to abortion during ascending placentitis and continues to rise until abortion occurs (Canisso et al., 2014; Coutinho da Silva et al., 2013). Treatment should lead to a decrease in SAA if the pregnancy is effectively preserved, and persistent elevation indicates a high likelihood of impending abortion (Canisso et al., 2014).

To avoid misdiagnosis of gestational disorders, it is essential to establish reference values that account for normal age-related variation in analytes. In Spanish Purebred mares, older individuals showed a pronounced increase in ACTH and CORT secretion, accompanied by altered patterns of APPs. Without age-adjusted benchmarks, physiological changes during pregnancy risk being mistaken for pathology, potentially leading to unnecessary interventions such as excessive monitoring, prolonged hospitalization, or immobilization. Defining specific reference ranges including minimum and maximum thresholds could improve differentiation between normal and pathological pregnancies, particularly in cases where inflammation is an early risk factor. Incorporating maternal age into diagnostic protocols may enhance clinical decision-making and improve outcomes in equine reproductive care. The understanding of the factors that cause age-related changes and how they should be managed clinically is evolving.

The extent to which hormonal and innumological changes with age are deemed “normal aging” vs “endocrine and innunological disease” could also be explored in greater depth, and specifically regard to the reproductive performance.

## 6 Limitations

One significant limitation of this study is the design of the non-pregnant control group, which was sampled at a single point during the breeding season. This lack of repeated measurements hinders longitudinal comparisons and makes it difficult to discern whether the physiological changes observed in pregnant mares were driven by gestation, the passage of time, or seasonal influences. Although the control animals were age-matched and sampled within the same reproductive window, the absence of serial data reduces the strength of conclusions regarding temporal dynamics.

Another limitation of this study is the absence of seasonal controls, which makes it difficult to separate pregnancy-related changes from those potentially driven by environmental conditions. Although baseline data were collected, the study was conducted during the summer months in Spain, a period characterized by high temperatures and increased insect activity. These seasonal factors may have influenced physiological responses—particularly the rise in CORT observed between 5 and 8 mo., which could reflect heat stress or insect irritation rather than gestation itself. Future studies should account for seasonal variation to better isolate the effects of pregnancy on endocrine and inflammatory markers.

## 7 Conclusions

The present study demonstrates that successful pregnancy in healthy mares is accompanied by activation of the HPA axis and the synthesis of multiple proinflammatory biomarkers, driven by a complex array of physiological stimuli. The trend in certain inflammatory markers (SAA, CRP) and the endocrine balance of the adrenal axis (ACTH, CORT), with related correlations, allow their role to be diversified according to age of mares. In fact, significant correlations between SAA and ACTH were observed exclusively in the older group.

These findings suggest that inflammatory and endocrine responses to pregnancy are amplified with advancing age, providing a useful set of exploration data for future research. Hence, further longitudinal studies in both animals and humans are warranted to better understand the mechanisms underlying HPA axis modulation and inflammatory pathways during gestation.

## Ethics approval

All methods and procedures used in the present study followed the guidelines of Spanish law (RD 37/2014) that regulates the protection of animals used for scientific purposes. The Animal Ethics Committee for the Care and Use of Animals of the CEU-Cardenal Herrera University (Spain) concluded that the proposed study did not need ethical approval, since this experiment was part of the clinical evaluation of the animals at this stage of their cycle.

## Data availability statement

The data that support this study will be shared upon reasonable request to be corresponding author.

## Funding

This research received no external funding.

## Informed consent statement

Informed consent was obtained from the owners of all subjects involved in the study.

## CRediT authorship contribution statement

**Deborah La Fauci:** Writing – review & editing, Data curation. **Pietro Medica:** Supervision, Formal analysis. **Esterina Fazio:** Writing – original draft, Investigation, Conceptualization. **Giuliana Barbiera:** Visualization, Supervision. **Maria Gemma Velasco-Martinez:** Validation. **Katiuska Satué:** Writing – original draft, Validation, Resources, Project administration, Methodology, Investigation, Conceptualization.

## Declaration of competing interest

The authors declare that they have no known competing financial interests or personal relationships that could have appeared to influence the work reported in this paper.
